# Coexistence of the Oxazolidinone Resistance–Associated Genes *cfr* and *optrA* in *Enterococcus faecalis* From a Healthy Piglet in Brazil

**DOI:** 10.3389/fpubh.2020.00518

**Published:** 2020-09-24

**Authors:** Lara M. Almeida, Anthony Gaca, Paulo M. Bispo, François Lebreton, Jose T. Saavedra, Rafael A. Silva, Irinaldo D. Basílio-Júnior, Felipe M. Zorzi, Pedro H. Filsner, Andrea M. Moreno, Michael S. Gilmore

**Affiliations:** ^1^Institute of Pharmaceutical Sciences, Federal University of Alagoas, Maceió, Brazil; ^2^Department of Clinical and Toxicological Analyses, Faculty of Pharmaceutical Sciences, University of São Paulo, São Paulo, Brazil; ^3^Department of Ophthalmology and Department of Microbiology, Harvard Medical School, Boston, MA, United States; ^4^School of Veterinary Medicine and Animal Science, University of São Paulo, São Paulo, Brazil

**Keywords:** oxazolidinones, resistance, *Enterococcus faecalis*, *cfr* gene, *optrA* gene, livestock

## Abstract

Oxazolidinones are one of the most important antimicrobials potentially active against glycopeptide- and β-lactam-resistant Gram-positive pathogens. Linezolid—the first oxazolidinone to be approved for clinical use in 2000 by the US Food and Drug Administration—and the newer molecule in the class, tedizolid, inhibit protein synthesis by suppressing the formation of the 70S ribosomal complex in bacteria. Over the past two decades, transferable oxazolidinone resistance genes, in particular *cfr* and *optrA*, have been identified in Firmicutes isolated from healthcare-related infections, livestock, and the environment. Our goals in this study were to investigate the genetic contexts and the transferability of the *cfr* and *optrA* genes and examine genomic features, such as antimicrobial resistance genes, plasmid incompatibility types, and CRISPR-Cas defenses of a linezolid-resistant *Enterococcus faecalis* isolated in feces from a healthy pig during an antimicrobial surveillance program for animal production in Brazil. The *cfr* gene was found to be integrated into a transposon-like structure of 7,759 nt flanked by IS*1216E* and capable of excising and circularizing, distinguishing it from known genetic contexts for *cfr* in *Enterococcus* spp., while *optrA* was inserted into an Inc18 broad host-range plasmid of >58 kb. Conjugal transfer of *cfr* and *optrA* was shown by filter mating. The coexistence of *cfr* and *optrA* in an *E. faecalis* isolated from a healthy nursery pig highlights the need for monitoring the use of antibiotics in the Brazilian swine production system for controlling spread and proliferation of antibiotic resistance.

## Introduction

Few drugs remain available for treating infections caused by antibiotic-resistant bacteria. Oxazolidinone antimicrobials, including linezolid and tedizolid, are among the few last-line therapies effective for multidrug-resistant (MDR) Gram-positive pathogens. Linezolid inhibits protein synthesis by targeting the peptidyl transferase center of the 50S subunit of bacterial ribosomes, blocking the binding of aminoacyl-tRNA to the A-site of the peptidyl transferase center (PTC), and also affecting the positioning of fMet-tRNA at the P-site, which prevents formation of the initiation complex (1–4). Over the past two decades, however, mutations in domain V of the 50S ribosomal subunit of 23S rRNA or in the ribosomal proteins L3 and L4 (5, 6) and the transferable resistance genes *cfr, optrA*, and *poxtA* (7–9) have driven the spread of oxazolidinone resistance in Gram-positive bacteria in healthcare and animal agriculture settings.

The spread of the multiresistance gene *cfr* has raised concern since its first report in a bovine *Staphylococcus sciuri* isolate (10). The *cfr* gene initially reported occurred in *Enterococcus* spp. from healthcare-related infections in Thailand (11) and from livestock in China (12). So far, a BLASTn search of the GenBank database identifies 3 *cfr* homologs in enterococci. While *cfr* has been found in both human and animal isolates of *Enterococcus faecalis* (Thailand, China) (11–13), *Enterococcus faecium* (Italy, Ireland, U.S.) (14–16), *Enterococcus casseliflavus*, and *Enterococcus thailandicus* (China) (17), the *cfr*(B) variant has been detected only in clinical isolates of *E. faecalis* (Japan) (18) and *E. faecium* (U.S., Germany, Netherlands) (19, 20). The most recently described *cfr*(D) variant has only four entries so far recorded in NCBI's databases, all *E. faecium* (France, Ireland, Netherlands) (21).

A Cfr-mediated adenosine modification A2503 in the PTC of 23S rRNA, which confers resistance to the oxazolidinone, phenicol, lincosamide, pleuromutilin, streptogramin A, and 16-member-ring macrolide antimicrobials (22), was until 2015 the only known transferable oxazolidinone resistance mechanism. Since then, the ATP-binding cassette (ABC)-F protein OptrA (23) has also been identified as conferring resistance to oxazolidinones, including the newer molecule in the class, tedizolid (24). *optrA* was identified in both *E. faecalis* and *E. faecium* of human and animal origins (8, 25, 26), as well as in *E. thailandicus* and *Enterococcus gallinarum* isolated from hospitals in China (27). Elsewhere in Asia (18, 28), Europe (29, 30), and America (16, 31), *optrA* has been found in *E. faecalis* and *E. faecium* of both human and animal origins. In Africa, *optrA*-positive *E. faecalis* isolated from humans (32), urban wastewater (33), and food-producing animals (34) were also reported. The *cfr* and *optrA* genes can be either plasmid or chromosomally encoded, and the co-location of both in the same plasmid has already been described in a porcine *Staphylococcus sciuri* isolate in China (35) and in *E. faecium* and *E. faecalis* recovered from hospitalized patients and livestock from Europe and the US (14–16).

In this study, we investigated the genetic contexts and the transferability of the *cfr* and *optrA* genes from the linezolid-resistant (LR) *E. faecalis* strain L9 (CP018004.1), which was isolated from a rectal swab collected from a healthy piglet in a surveillance study of antimicrobial susceptibility in Brazil's swine production system (36). Antimicrobial resistance genes, plasmid incompatibility types, epidemiology, and CRISPR-Cas (Clustered Regularly Interspaced Short Palindromic Repeat) defenses of LR *E. faecalis* L9 were also examined. Whole-genome sequencing (WGS) analysis revealed the presence of *cfr* associated with a transposable element capable of excision and formation of an intracellular circular intermediate flanked by IS*1216E* (CP041775.1), which is different from all previously known genetic contexts in *Enterococcus* spp. from human and animal sources. Further, the core *araC*-hp-*optrA* was found to be inserted into a conjugative Inc18 broad host-range plasmid of >58 kb (CP041776.1) in LR *E. faecalis* L9.

## Materials and Methods

### Bacterial Isolation

LR *E. faecalis* L9 comes from a collection of 13 LR *E. faecalis* (linezolid MIC of 8 mg/L) that were screened from 245 MDR *E. faecalis* isolated from rectal swabs from healthy piglets (45 days old) in different states of Brazil (36). These 13 *optrA*-positive *E. faecalis*, epidemiologically unrelated (ST29, ST330, ST591, ST710, ST711), were recovered from different pigs found to be distributed in 6 out of the 7 states chosen for sample collection. Three LR *E. faecalis* isolated in the same state (DF) harbored both *optrA* and *cfr* (ST591 and ST29), but conjugal transfer of these resistance genes to an enterococcal recipient was achieved only using the ST29 *E. faecalis* strain L9 as donor in our previous filter mating assays. Therefore, here we investigated the mobile element types that enabled horizontal transfer of *cfr* and *optrA*.

### Whole-Genome Sequencing and Data Analysis

LR *E. faecalis* L9 was grown in brain heart infusion (BHI) broth at 37°C (24 h). Genomic DNA was isolated using the QIAGEN DNeasy Blood & Tissue Kit, and quantified using Qubit dsDNA HS. Sequencing libraries were prepared with the Illumina Nextera XT DNA kit and sequenced on a MiSeq instrument (Illumina Inc., USA) at the Massachusetts Eye and Ear Infirmary (MEEI) Ocular Genomics Institute, as 250 nt paired-end reads. *De novo* assembling was performed using CLC Genomics Workbench 8.0.3. For genome annotations, both the RAST server (Rapid Annotation using Subsystem Technology) and the Prokaryotic Genome Annotation Pipeline (NCBI PGAP) were used. Genome data analysis was performed using BLAST (http://blast.ncbi.nlm.nih.gov/Blast.cgi) and Center for Genomic Epidemiology (http://www.genomicepidemiology.org) online tools. ResFinder (https://cge.cbs.dtu.dk/services/ResFinder/) was used to identify acquired antimicrobial resistance genes, and PlasmidFinder (https://cge.cbs.dtu.dk/services/PlasmidFinder/) was used to determine plasmid incompatibility types. For detection of the oxazolidinone resistance determinants, LRE-Finder (https://cge.cbs.dtu.dk/services/LRE-finder/) was used as well. Multilocus sequence typing (MLST) loci were assigned by the MLST database (https://pubmlst.org/efaecalis/), and the presence of CRISPR-*cas* defenses was identified by CRISPRfinder (https://crisprcas.i2bc.paris-saclay.fr).

### Filter Mating Assay

Conjugation by filter mating as described previously by Jaworski and Clewell (37) was performed using LR *E. faecalis* L9 as donor, and the *E. faecalis* strain OG1RF as recipient. Donor and recipient were grown overnight in BHI broth at 37°C. One milliliter from donor culture plus 1 ml from recipient culture were inoculated in 3 ml of phosphate buffered saline (PBS) solution, filtered through a sterile 25-mm-diameter, 0.22-μm-pore-size membrane filter, and subsequently incubated on BHI agar at 37°C for 24–48 h. PBS (5 ml) was used to wash the filters, and 500 μl of this solution was spread on BHI agar plates (100 × 15 mm Petri plates) containing 25 μg/ml of fusidic acid, 25 μg/ml of rifampicin to select for the OG1RF chromosomal markers, and 25 or 10 μg/ml of chloramphenicol (CHL) to select for oxazolidinone and phenicol resistance genes; linezolid (LZD) (4 μg/ml) instead of chloramphenicol was also tested to select for *cfr* and *optrA*. Conjugation efficiency (CFU/ml of transconjugants per CFU/ml of donors) was calculated as previously described (38). PCR using primer sets specific for *optrA, cfr, poxtA, fexA*, and *cat* genes (36) and Sanger sequencing were carried out to detect these resistances in OG1RF transconjugants. Minimum inhibitory concentrations (MIC's) of chloramphenicol, florfenicol, linezolid, and tedizolid were determined by broth microdilution testing according to the guidelines of the Clinical Laboratory Standards Institute (CLSI). *E. faecalis* ATCC 29212 was used as a control for antimicrobial susceptibility testing.

## Results and Discussion

*Enterococcus faecalis* is a commensal bacterium of the gut microbiota of humans and various animal species and also a cause of infections in critically ill patients (39, 40). Besides being an important hospital pathogen, *E. faecalis* has emerged as a potential reservoir of oxazolidinone resistance genes in animal agriculture settings worldwide (8, 31, 34, 36, 41). It is of substantial concern that antibiotics used in food-producing animals may be selecting for the proliferation of MDR *E. faecalis* lineages in which *cfr* and *optrA* coexist. Cfr rRNA methyltransferase confers resistance to six important antimicrobial classes that target the 50S ribosomal subunit (22), while the ATP-binding cassette (ABC)-F protein OptrA confers resistance to phenicol and oxazolidinone, including resistance to the new oxazolidinone tedizolid (23). The spread of *cfr* and *optrA* inter-species/genera has been driven by plasmids containing other important resistance determinants (14–16, 35). Therefore, oxazolidinone resistance can be co-selected by antimicrobials that have been largely used in swine production, such as phenicol, macrolide, lincosamide, and pleuromutilin.

### Antimicrobial Resistance Determinants of LR *E. faecalis* L9

ResFinder identified that LR *E. faecalis* L9 carries the *lsa*(A) gene, which is responsible for intrinsic LS_A_P resistance in *E. faecalis*, and acquired resistance genes for aminoglycoside (*str*), phenicol *fex*(A), phenicol and oxazolidinone (*optrA*), and tetracycline [*tet*(L), *tet*(M), *tet*(S)], in addition to the multiresistance gene *cfr*. LRE-Finder confirmed the presence of *cfr* and *optrA* (CP041775.1 and CP041776.1, respectively), but the phenicol–oxazolidinone–tetracycline resistance gene *poxtA* was not found. 23S rRNA mutations were not detected in LR *E. faecalis* L9, nor were they identified in ribosomal protein genes *rplC, rplD*, and *rplV* (L3, L4, and L22, respectively).

### Genetic Context of *cfr* in the Porcine LR *E. faecalis* Isolate L9

**Figure 1:**
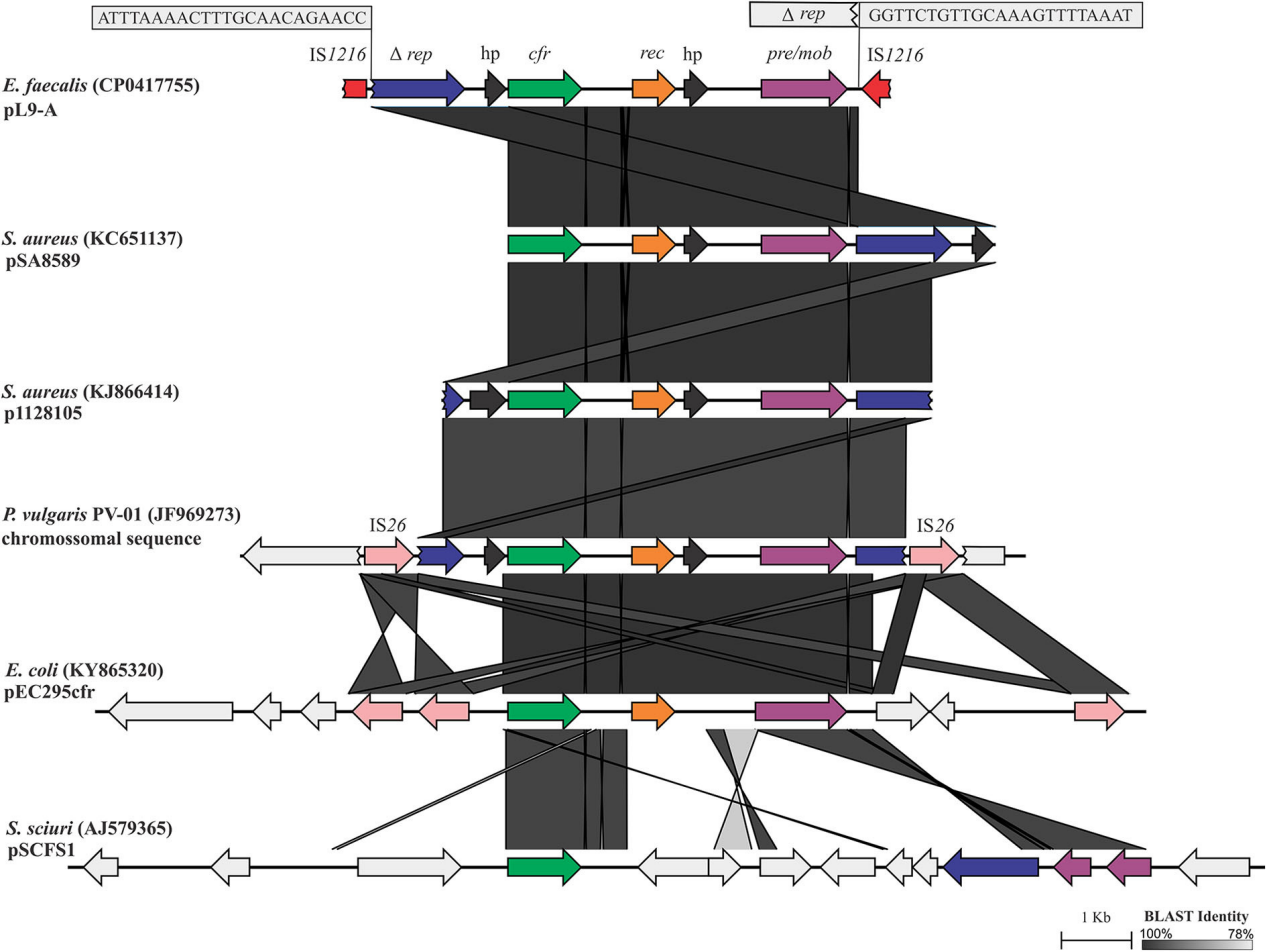
Linear comparison of the 7,759-bp *cfr*-carrying DNA segment pL9-A (CP041775.1) generated by EasyFig. The boxes zoom in on the 23-bp inverted repeats (IR) at the ends of an IS*1216* that was inserted into *repUS18*. The 6,956-bp segment between the IRs shows high DNA identity to the corresponding stretches in pSA8589 (KC651137), p1128105 (KJ866414), and *P. vulgaris* PV-01 chromosomal sequence (JF969273). Alignment of these sequences revealed only a deletion of 7 bp in *repUS18* from pL9-A and an insertion of 10 bp in the hypothetical protein on the flank 5′ of *cfr* in p1128105, which is represented by the slightly lighter shade of identity over this region.

A *cfr*-carrying DNA segment of 7,759 nt, pL9-A (CP041775.1), was found to be inserted into LR *E. faecalis* L9 (Figure 1). The *cfr* gene was flanked upstream by the Tn*554*-related Δ*tnpB* gene. Further upstream of Δ*tnpB*, a gene coding for RepUS18 was detected that was disrupted by the integration of an IS*1216E*. The *repUS18* gene is often found in Inc18 broad host-range plasmids, which have been related to antimicrobial resistance gene transfer in enterococci. Downstream, the *cfr* gene was flanked by a recombinase *rec* gene, a gene coding for a hypothetical protein, and a plasmid recombination/mobilization *pre*/*mob* gene. *In silico* predictions indicated that the IS*1216E*-flanked segment pL9-A could excise and exist within the cell as a non-replicating circular intermediate in LR *E. faecalis* L9, which was confirmed by PCR and Sanger sequencing using the primers 5′AGGTTTAGAATAATCTCCCGA3′ and 5′GCTGACAACATATCTAATATCTCAA3′.

pL9-A possesses 100% DNA identity over 93% of its length to a chromosomal DNA sequence from *Proteus vulgaris* PV-01 (JF969273) isolated from a pig nasal swab in China (42), and 99.98% and 99.83% DNA identity to the pSA8589 and p1128105 from *Staphylococcus aureus* 1900 (43) and *S. aureus* 1128105 (44) of human origin in the US (KC561137 and KJ866414, respectively), highlighting its very broad-range horizontal transfer capabilities. IS*6* insertion sequence family elements, which have been commonly associated with antibiotic resistance genes, appear to be also involved in transposition events of the core *cfr-rec-pre/mob* in Gram-positive and Gram-negative bacteria.

The *cfr*-carrying mobile element pL9-A was distinctly different from all known genetic contexts of *cfr* in *Enterococcus* spp. from human and animal sources. The similarity of pL9-A to the *cfr*-carrying segments previously identified in bacteria of other genera indicates that it has most likely been acquired horizontally from other bacteria or, alternatively, could be intrinsic in some lineages of *E. faecalis* and then transferred to other bacteria. The IS*1216E* element appears to be involved in the acquisition and dispersal of pL9-A in LR *E. faecalis* L9. In Brazil, the *cfr* gene has been reported to date only in an ST398 MSSA strain of human origin (45) in a genetic context other than that observed in the porcine LR *E. faecalis* isolate L9.

### Plasmid-Borne *optrA*-Carrying Partial Sequence (pL9) in the Porcine LR *E. faecalis* Isolate L9

**Figure 2:**
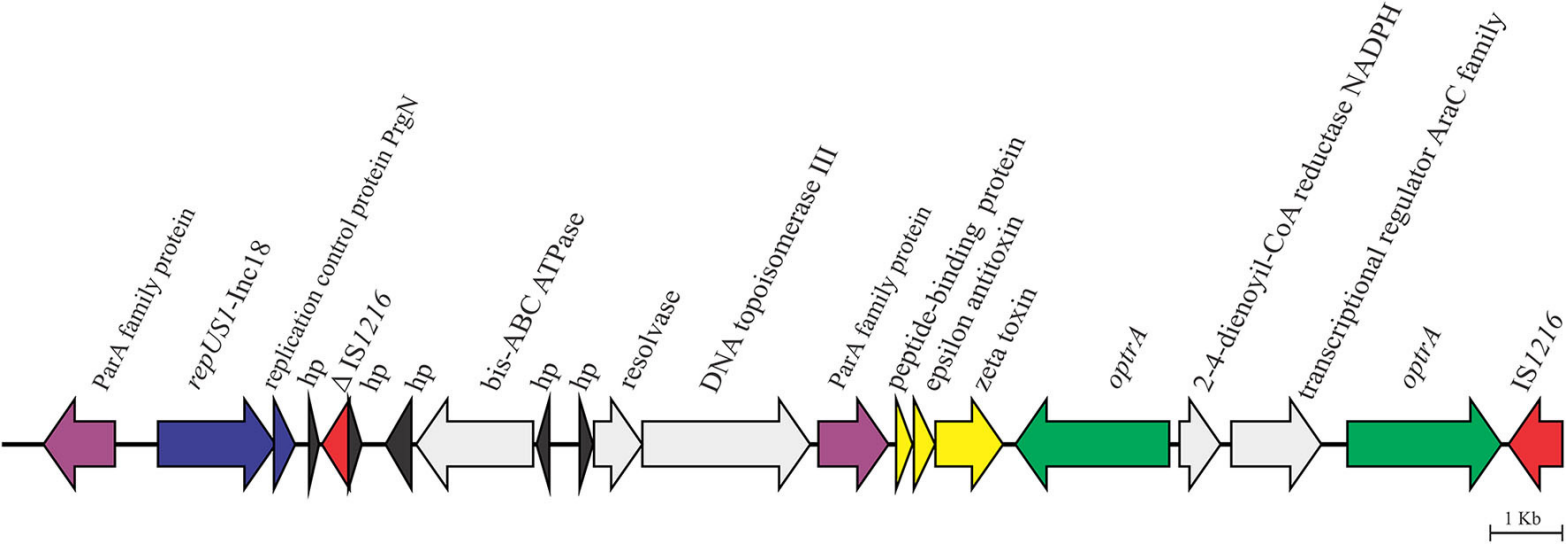
Genetic context of the 58,593-bp *optrA*-carrying partial sequence pL9 (CP041776.1) in the porcine LR *E. faecalis* isolate L9.

We recently reported that the core *araC*-hp-*optrA* of 3,453 nt in length, which was composed of genes coding for a hypothetical protein and an AraC family transcriptional regulator at the 5′ of *optrA*, was inserted upstream of an IS*1216E* element into a plasmid of >58 kb, which was not closed during *de novo* assembly of the high quality draft sequence (CP041776.1) (36). On the flank 5′ of the core *araC*-hp-*optrA*, LR *E. faecalis* L9 showed *in silico* a duplication of *optrA*, which was confirmed by PCR and Sanger sequencing using the primers 5′TTGAGTGAAATACCTGTGCG3′ and 5′TGATGGTAATATGGTGTTGGAA3′. Further analysis of pL9 showed the presence of genes coding for the zeta–epsilon–delta (ω-ε-ζ) toxin–antitoxin (TA) system upstream of the duplication of *optrA* (Figure 2). The ω-ε-ζ TA module, a post-segregational killing system which acts at cell division eliminating progeny that fails to inherit plasmid copy, has been found in various MDR Gram-positive bacteria, including the *cfr*-carrying conjugative plasmids pW9-2 from *E. faecalis*, and pW3 and p3-38 from *E. thailandicus* isolated from sewage in swine farm contexts in China (12) and pEF12-0805 from *E. faecium* isolated from human blood in Italy (14). pL9 harbored a gene coding for the plasmid replication protein *repUS1*, which is found in Inc18 broad host-range plasmids. On the 5′ flank of *repUS1*, a 912-nt open reading frame (ORF) for the partitioning protein ParA which mediates plasmid segregation was found. These mechanisms ensure the maintenance of plasmids that exist in low-copy numbers in a bacterial population, such as Inc18 family plasmids. At the 3′ flank of *repUS1*, a 288-nt ORF for the replication control protein PrgN is present. Upstream to this region, an 1,494-nt ORF for ATP-binding cassette domain-containing protein came to our attention due to the very few entries so far recorded in NCBI's databases, as it only matches nucleotide sequences from 6 *Lactococcus garvieae*, which causes fatal hemorrhagic septicaemia in fish (South Korea and Japan), and 1 *Lactococcus petauri* isolated from human feces in China. This ORF codes for the ATP-binding cassette domain-containing protein (*E. faecalis* WP_155282194.1), which has 81.74% DNA identity over 99% of its length to the *Lactococcus* ABC-F-type ribosomal protection protein (WP_019291880.1).

pL9 was found to be inserted into a conjugative Inc18 plasmid of >58 kb. Inc18 broad host-range plasmids have been associated with a variety of antibiotic resistances in enterococci, including the high-level *vanA* glycopeptide resistance carried by Tn*1546*, which can be transferred to MRSA lineages (46). Inc18 plasmids can play a crucial role in the oxazolidinone resistance emergence, as they are widespread in enterococci, streptococci, and staphylococci in both clinical and environmental settings (47, 48). Moreover, most Inc18 plasmids carry locus coding for stabilization systems, such as the post-segregation killing (PSK) system (49), which has already been implicated in the persistence of the Tn*1546*-mediated *vanA* resistance in *E. faecium* (50). The presence of ORF's adjacent to *optrA* in pL9 that matched few or no DNA sequences available in GenBank indicates that further investigation is required to understand how new conjugative Inc18 plasmid mosaics are evolving and how that might favor the spread of oxazolidinone resistance in animal agriculture settings.

### Transferability of *cfr, optrA*, and Other Resistance Determinants in LR *E. faecalis* L9

Filter mating assays were carried out to determine the potential for conjugal transfer of *cfr* and *optrA* at different CHL and LZD concentrations (Table 1). *optrA*/*fexA*/*tet*(S)-carrying OG1RF-L9 transconjugants were selected at a frequency of 4 × 10^−7^ transconjugant cells per donor cell using 25 μg/ml CHL, but conjugation experiments failed to transfer *cfr* at 25 μg/ml CHL. Decreasing CHL concentration from 25 to 10 μg/ml, countless small colonies of *optrA*/*cfr*/*fexA*/*tet*(S)-carrying OG1RF-L9 transconjugants could be selected. Linezolid could select only countless small colonies of *optrA*-positive OG1RF-L9 transconjugants; no *cfr*-positive OG1RF-L9 transconjugant was obtained, indicating that *optrA* is responsible for linezolid resistance, and *cfr*, for a lower-level chloramphenicol resistance phenotype in LR *E. faecalis* L9.

**Table 1:** Conjugation efficiency of *cfr* and *optrA* from *E. faecalis* L9 to *E. faecalis* OG1RF transconjugants.

**Recipient strain^**a**^**	**Conjugation efficiency^**b**^**	**Resistance genes^**c**^**	**MIC (μg/ml)**			
			**LZD**	**TZD**	**CHL**	**FFC**
OG1RF-L9 (25 μg/ml CHL)	4 × 10^−7^	*optrA, fex*(A), *tet*(S)	8	0.5	128	64
OG1RF-L9 (10 μg/ml CHL)	ND	*optrA, fex*(A), *tet*(S), *cfr*	8	0.5	128	64
OG1RF-L9 (8 μg/ml LZD)	ND	*optrA, fex*(A), *tet*(S)	8	0.5	128	64
OG1RF-L9 (4 μg/ml LZD)	ND	*optrA, fex(A), tet(S)*	8	0.5	128	64

a *Graded levels of CHL and LZD were tested to select for oxazolidinone and phenicol resistance genes, in addition to 25 μg/ml fusid acid, and 25 μg/ml rifampicin to select for the OG1RF chromosomal markers*.

b *Conjugation efficiency corresponds to the number of CFU transconjugants per CFU donors; ND (not determined): only small bacterial colonies (countless) were obtained, all optrA/fexA-positive*.

c *optrA could be efficiently transferred by conjugation at a frequency of 4 × 10^−7^ per donor cell using 25 μg/ml of CHL as previously reported (36), and also decreasing CHL concentration or using LZD instead of CHL*.

Tn*558*, which harbors the chloramphenicol/florfenicol efflux MFS transporter *fexA* gene and the tetracycline resistance ribosomal protection gene *tet*(S), was also transferred to OG1RF-L9 in a yet to be determined genetic context, as pL9 could not be closed during *de novo* assembly. A 204-nt fragment of a gene for a conjugal transfer protein 5′ of *tet*(S) that appears to be involved in its mobilization is identical to homologs occurring in Firmicutes as identified in a BLASTn search. A 210 nt ORF 3′ flank to Tn*558* matched a gene coding for the replication-associated protein RepB identified in *Listeria monocytogenes* (KY613776.1 and KY613741.1) isolated from food in Canada, in *Carnobacterium divergens* (LT984411.1) from beef carpaccio in France, and in an *optrA*-carrying conjugative plasmid from the *Enterococcaceae* strain E508 (MK425645.1) in China. Another 606-nt ORF encoding a hypothetical protein at the extreme 3′ end of *repB* also matched ORF's from the *L. monocytogenes, C. divergens*, and *Enterococcaceae* isolates mentioned above, and the *optrA*-carrying *Enterococcus avium* isolate C674 (MH018573.1) from an asymptomatic healthy human in China. At 5′, Tn*558* is flanked by a 210-nt ORF for a hypothetical protein that only matches sequences from the *optrA* gene cluster from *E. avium* C674 and pStrcfr from *Streptococcus suis* S10 (KF129409.1). Further upstream, a 1,272-nt ORF for a Y-family DNA polymerase is present, but no match was found for this nucleotide sequence.

### Bacterial Immunity of *cfr/optrA*-Carrying *E. faecalis* L9

Genome defenses for porcine LR *E. faecalis* L9 were investigated using CRISPRfinder. Clustered, regularly interspaced short palindromic repeat (CRISPR) loci provide an important defense against parasitic mobile element entry. MDR, hospital-adapted enterococcal lineages lack CRISPR defenses, which are thought to enhance the facility with which they acquire antibiotic resistances on mobile elements (39, 51). A CRISPR-related loci consisting of 9 spacers and direct repeat sequences of 36 bp was found in the L9 chromosome (1,708,044 to 1,708,673 bp), but genes coding for Cas proteins were not identified. A BLASTn search revealed that the L9 CRISPR-related loci possess 95.12% DNA identity over 100% of its length to the corresponding chromosomal DNA sequence from *E. faecalis* SGAir0397 (CP039434.1), which was recovered from air in Singapore. L9 CRISPR spacer sequences only matched to sequences from *E. faecalis* FDAARGOS_324 (CP028285.1) isolated from a human eye in the US, and the cyanobacterium *Geminocystis* sp. isolate NIES-3708 (AP014815.1) from Japan, besides *E. faecalis* SGAir0397. LR *E. faecalis* L9 lacked the *E. faecalis* CRISPR1 locus (a CAS-TypeIIA cluster consisting of Csn2_0_IIA, Cas2_0_I-II-III, Cas1_0_II, Cas9_1_II) typically located between genes EF0672 and EF0673 (51).

To the best of our knowledge, this is the first report of the coexistence of *optrA* and *cfr* in a bacterial isolate in Brazil. The fact that LR *E. faecalis* L9 came from a pool of 13 LR *E. faecalis* collected from healthy piglets in swine herds distributed across 7 Brazilian States highlights the need for monitoring the use of antibiotics in the country's swine production system in order to preserve the few remaining last-line antibiotics to treat infections caused by MDR pathogens.

## Data Availability Statement

The datasets presented in this study can be found in online repositories. The names of the repository/repositories and accession number(s) can be found below: https://www.ncbi.nlm.nih.gov/genbank/, CP018004.1, https://www.ncbi.nlm.nih.gov/genbank/, CP041775.1, https://www.ncbi.nlm.nih.gov/genbank/, CP041776.1.

## Ethics Statement

The study was approved by the Ethics Committee of Faculdade de Medicina Veterinária e Zootecnia- Universidade de São Paulo, under number CEUA N.8026060214.

## Author Contributions

LA and MG planned the study and wrote the manuscript with suggestions from AG, PB, and FL. PF and AM provided a collection of 245 MDR *E. faecalis* strains isolated from swine. LA, JS, RS, and IB-J carried out the experimental work and genome sequencing. LA, FZ, AG, PB, and FL contributed to the bioinformatic analyses. All authors contributed to the article and approved the submitted version.

## Conflict of Interest

The authors declare that the research was conducted in the absence of any commercial or financial relationships that could be construed as a potential conflict of interest.
